# Perceived and endocrine acute and chronic stress indicators in fibromyalgia syndrome

**DOI:** 10.1038/s41598-024-76635-z

**Published:** 2024-12-16

**Authors:** Eva Beiner, Michelle Hermes, Julian Reichert, Kristian Kleinke, Stephanie Vock, Annette Löffler, Leonie Ader, Andrei Sirazitdinov, Sebastian Keil, Tim Schmidt, Anita Schick, Martin Löffler, Michael Hopp, Christian Ruckes, Jürgen Hesser, Ulrich Reininghaus, Herta Flor, Wolfgang Eich, Hans-Christoph Friederich, Jonas Tesarz

**Affiliations:** 1 Department of General Internal Medicine and Psychosomatics, Heidelberg University, Heidelberg, Germany; 2 Department of Psychology, University of Siegen, Siegen, Germany; 3 Institute of Cognitive and Clinical Neuroscience, Medical Faculty Mannheim, Central Institute of Mental Health, Heidelberg University, Mannheim, Germany; 4 Department of Psychiatry, Psychotherapy and Psychosomatics, Faculty of Medicine, RWTH Aachen, Aachen, Germany; 5 Department of Psychosomatic Medicine and Psychotherapy, Medical Faculty Mannheim, Central Institute of Mental Health, Heidelberg University, Mannheim, Germany; 6 Department of Public Mental Health, Medical Faculty Mannheim, Central Institute of Mental Health, Heidelberg University, Mannheim, Germany; 7 Data Analysis and Modeling in Medicine, Mannheim Institute for Intelligent Systems in Medicine (MIISM), Medical Faculty Mannheim, Heidelberg University, Heidelberg, Germany; 8 CZS Heidelberg Center for Model-Based AI, Heidelberg University, Heidelberg, Germany; 9 Central Institute for Computer Engineering (ZITI), Heidelberg University, Heidelberg, Germany; 10 Interdisciplinary Center for Scientific Computing (IWR), Heidelberg University, Heidelberg, Germany; 11 Clinical Psychology, Department of Experimental Psychology, Heinrich Heine University Düsseldorf, Düsseldorf, Germany; 12 Interdisciplinary Center for Clinical Trials, Johannes Gutenberg University Medical Center Mainz, Mainz, Germany; 13 DZPG (German Centre for Mental Health – Partner Site Heidelberg/ Mannheim/ Ulm), Heidelberg, Germany; 14 Centre for Epidemiology and Public Health, King’s College London, London, UK; 15 Health Service and Population Research Department, Institute of Psychiatry, Psychology & Neuroscience, King’s College London, London, UK; 16 Scientific Center for Neuropathic Pain Aachen SCN AACHEN, Aachen, Germany; 17 Department of Psychosomatic Medicine and Psychotherapy, University Medical Center of the Johannes Gutenberg University Mainz, Mainz, Germany; 18 Internal Medicine II, University Hospital Heidelberg, Im Neuenheimer Feld 410, 69120 Heidelberg, Germany

**Keywords:** Fibromyalgia Syndrome, Perceived stress, Endocrine stress indicators, Biomarkers, Medical research

## Abstract

Fibromyalgia syndrome (FMS) is a chronic disorder characterized by widespread musculoskeletal pain, fatigue and tenderness and closely associated with high levels of stress. FMS is therefore often considered a stress-related disease. A comparative study was conducted with 99 individuals diagnosed with FMS and a control group of 50 pain-free individuals. Stress indicators were classified into three categories: perceived stress assessed using the Perceived Stress Scale, and daily average salivary cortisol and hair cortisol concentrations as indicators of acute and chronic stress levels related to the hypothalamic-pituitary-adrenal axis. Analysis of variance and covariance were used to identify group differences and the influence of covariates age, sex, and body mass index. Correlational analyses further elucidated the relationship between stress indicators and clinical symptoms. Participants with FMS reported significantly higher perceived stress levels than controls (*p* < .001, η_p_^2^ = 0.3), which were positively correlated with symptom burden (*r* = .41, *p* < .001). In contrast, there were no significant differences in the endocrinological stress indicators salivary and hair cortisol between the groups (*p* > .05), nor were these indicators associated with clinical symptoms. The study highlights the central role of perceived stress in FMS, whereas endocrinological indicators did not differentiate FMS from controls. This finding calls for a nuanced approach to clinical assessment and therapeutic interventions tailored to patients with FMS, emphasizing the management of perceived stressors.

**Supplementary Information:**

The online version contains supplementary material available at 10.1038/s41598-024-76635-z.

## Introduction

Fibromyalgia Syndrome (FMS) is a complex and multifaceted chronic pain disorder characterized by widespread musculoskeletal pain, fatigue, cognitive dysfunction, and sleep disturbances^1–4^. About 0.2–6.6% of the population is affected by FMS^5^, with a prevalence of 2.4–6.8% among women, though men are also affected^6^. The pathophysiology of FMS remains a topic of intense research, with recent studies reporting the presence of autoantibodies against dorsal root ganglia and discussing the possibility of small fiber pathology^7, 8^. Additionally, there is strong evidence indicating a significantly increased prevalence of early childhood stress exposure and psychological trauma among individuals with FMS, highlighting the role of stress in the disorder^2, 3, 9, 10^. These findings suggest that altered stress reactivity might serve as a potential biopsychosocial link between the psychological, social, and biological aspects of FMS, making it a renewed focus of interest.

The body’s stress-regulation systems, such as the hypothalamic-pituitary-adrenal (HPA) axis and the sympathoadreno-medullary (SAM) axis, are crucial in the development of stress-related disorders and may influence the chronic pain and symptom amplification observed in FMS^11, 12^. Considering these insights, a comprehensive understanding of the interactions between these stress systems, the brain’s neural networks, and the complex physiological, psychological, and social factors is essential for advancing the diagnosis and treatment of FMS^3, 13, 14^. This multifaceted perspective underscores the critical importance of addressing stress, both acute and chronic, in the assessment and treatment of FMS.

Stress is a latent variable that can be measured through different approaches. Most approaches use either cortisol as an endocrine indicator of stress^15^ or questionnaires investigating the experience of perceived stress^16^. Cortisol is used as endocrine stress marker as it rises in response to the exposure to unpleasant stimuli, mental burden, acute demands, or illnesses that induce changes of the body’s homeostasis and activate the HPA-axis^15, 17^. After the activation of the HPA-axis, a cascade of hormones is released in response to the stressor, resulting in elevated cortisol levels to compensate the body’s stress reactivity to sustain homeostasis^15, 17, 18^.

Cortisol can be assessed through different methods. It can either be measured through urine, saliva, hair or through blood. During acute stress responses, one can detect elevated cortisol levels in both blood and saliva through single-time-point measurements. However, blood cortisol needs to be measured invasively, which poses a major barrier for many participants, while salivary cortisol can be collected non-invasively and more convenient. Although salivary cortisol is strongly influenced by circadian fluctuations throughout the day, which must be considered and several samples need to be collected per day, it offers a useful tool for cortisol measures^19–21^. Common parameters used to investigate salivary cortisol are the daily average cortisol (DAC), the increase in cortisol awakening response (CARi) or the area under the curve (AUC)^22–24^. However, these measurements prove disadvantageous when aiming to obtain a measure of long-term systemic cortisol production^19, 25^. When evaluating prolonged systemic cortisol production, hair cortisol offers distinct advantages. Due to easy sampling and reduced participant burden, hair cortisol can be easily determined retrospectively for a longer time period. Further, hair samples can easily be stored and transported at room temperature, whereas blood, saliva or urine measurements must be analyzed immediately or need to be frozen^26^. With approximately 1 cm growth rate per month, a hair sample of several centimeters can provide the information on cortisol over several months and can serve as an endocrine measure for long-term stress. Consequently, hair cortisol concentration has been suggested as a suitable indicator for assessing chronic stress levels^19, 26, 27^.

While an elevated stress experience in patients with FMS has been clinically suggested and documented in the literature^2, 9, 28–31^, there exists considerable inconsistency in the evidence regarding alterations in the endocrine indicators of HPA-axis functionality^11, 21, 32–35^. Findings on cortisol range from no differences in blood or hair cortisol concentrations^32, 36–38^, over higher levels of hair or blood cortisol^39, 40^ to hypocortisolism in FMS^34, 41, 42^. Further, the findings on stress perception associated with endocrine markers is also heterogenous, with some studies reporting significant associations for example between hair cortisol and perceived stress^37^, while others could not find this relationship^36^. These heterogenous findings may result from different potential reasons including variability in study designs and sample sizes, measurement methods (in blood, saliva, urine) and variability in the assay methods used to analyze these markers^20^. Further, cortisol measurements are influenced by multiple factors such as age, gender and menstrual cycle, physical activity, mental health, exposure to adverse events, ethnicity, or weight^9, 12, 20, 33, 43, 44^. Hence, there is currently a lack of consensus regarding the relationship between perceived stress levels in people with FMS and the endocrine indicators for acute and chronic stress associated with systemic cortisol production in the HPA-axis.

In this study, our primary aim is therefore to identify potential variations in perceived and endocrine indicators of stress in individuals with FMS including perceived stress, acute cortisol response and chronic cortisol levels. To explore these variations, we 1.) compared these three indicators of stress between individuals diagnosed with FMS and pain-free controls, and 2) investigated the associations between these stress indicators and clinical symptoms.

As chronic stress-associated conditions and chronic pain have been associated with a suppression of HPA-activity^10, 14, 32, 35^, we postulate that people with FMS will have subjectively elevated stress levels compared to pain-free controls, with reduced HPA-axis activity^32^, basal cortisol levels and hair cortisol levels. We also expect that more severe and more pronounced symptoms of FMS will result in higher subjective levels of stress and higher acute levels of salivary cortisol.

## Methods

This study was part of the PerPAIN study, a large multicenter study, with the goal to phenotype pain patients on a multilevel perspective, to identify subgroups of pain patients according to their pain characteristics. The project was funded by the German Federal Ministry of Education and Research. The study protocol was approved by the Ethics Research Committee II of the Faculty of Medicine, University of Heidelberg (2020–579 N) and was carried out in compliance with the Helsinki Declaration. For further details on the design of the underlying multicenter study see Beiner et al. 2022 ^45^. This study only used data of the baseline assessment of the PerPAIN study. Due to local data protection regulations, direct release of the data in the sense of accessibility is not possible. The possibilities for data availability must be clarified with the corresponding author on request in individual cases. The preregistration was uploaded on open science framework (OSF.IO/G5DU7)^46^.

### Study design

#### Recruitment and inclusion criteria

The PerPAIN study recruited a total of 346 participants, from which 320 suited and were screened for eligibility. Finally, 264 individuals participated in the PerPAIN study between April 2020 and August 2023, with 214 individuals suffering from chronic pain and 50 pain-free controls. The sample for this secondary data analysis was a subsample of the PerPAIN study. Patients were recruited from the pain clinic at the University Hospital Heidelberg, the affiliated hospital in Baden-Baden, as well as the Central Institute of Mental Health in Mannheim. Patients had to meet the inclusion criteria of suffering from chronic musculoskeletal pain for more than three months as a symptom of (1) non-specific chronic back pain, (2) osteoarthritis, (3) fibromyalgia syndrome, or (4) rheumatoid arthritis. Further inclusion criteria were the ability to see and use a mobile telephone (including with visual aids), age ≥ 18 years (no upper age limit), and the ability to provide informed consent.

For inclusion in this study, all participants were assessed by a study physician for the presence of FMS according to the 1990 or 2016 American College of Rheumatology (ACR) criteria^47–50^, be at least 18 years old, must have had symptoms for at least three months, and be able to give informed consent. Additionally, all participants were screened for the following exclusion criteria: secondary pain disorders, insufficient or unclear treatment of the underlying disease, pending application for retirement/pension, ongoing psychotherapy, severe pharmaceutically treated acute life-threatening physical comorbidity, physical comorbidity incompatible with study participation, severe mental disorder (inability to consent, suicidality, psychosis spectrum disorders), neurological comorbidities (e.g., epilepsy, traumatic brain injury, seizures, multiple sclerosis, neurodegenerative diseases), and pregnancy.

To be included in the control group, the participants had to be free of any acute or chronic pain and mentally and physically healthy at the time of the study, which was assessed by a trained study physician. A flow chart describing the inclusion process, as well as a procedure outline of the PerPAIN study can be found in the supplementary material (Appendix A and B respectively).

### Measurements

#### Stress indicators

To comprehensively map individual stress responses, we collected not only data on the level of perceived stress, but also daily cortisol profiles on two consecutive days to document the short-term activity of the HPA-axis as indicator for acute stress, and hair cortisol levels to document the long-term activity of the HPA-axis over the previous three months as an indicator for chronic stress.

##### Perceived stress

To measure the level of perceived stress, the German version of the Perceived Stress Scale (PSS)^51^ was used. The PSS measures perceived stress through ten items, asking about the participants’ feelings over the last month. Questions include, for example, “*In the last month*,* how often have you felt nervous and stressed?*”. Answers range from *“0 = Never*” to “*4 = Very Often*”. The sum score was calculated and used for the analysis. The questionnaire is recommended for phenotyping patients for large scale studies^16^.

##### Cortisol as acute stress indicator

As a measure of short-term activity of the HPA-axis, salivary cortisol was measured over two consecutive days, adhering to established recommendations for daily average cortisol (DAC)^22, 24^. Participants provided a total of 12 saliva samples—six samples per day. Sampling occurred immediately upon awakening, followed by 15 min, 30 min, and 60 min post-awakening. Additional samples were collected in the afternoon at 3 pm and in the evening at 8 pm, to cover the daily average of cortisol. Saliva samples were frozen (-20 °C) until start of analysis. All saliva samples were analyzed in the central laboratory and steroid laboratory of the University Hospital Heidelberg. The standard operating procedures were used, according to the instructions of the manufacturer. They were then centrifuged and analyzed with Liquid Chromatography Mass Spectrometry/Mass Spectrometry (LC-MS/MS) on a Waters Xevo TQ-S System. For the analysis, an average cortisol parameter over both days was calculated^22, 24^.

##### Chronic cortisol indicator

Hair cortisol was analyzed to measure the long-term activity of the HPA-axis over a 3-month period. To achieve this, participants had to donate a hair strand of at least 3 cm length. The hair sample was collected from the posterior region of the scalp, with the strand being cut as proximate to the scalp as feasible. All hair samples were analyzed at Dresden University of Technology (TU Dresden). After hair sample extraction, the samples were packed in aluminum foil to be stored dry and dark. The analysis of cortisol was done by using Liquid Chromatography Mass Spectrometry/Mass Spectrometry (LC-MS/MS) method according to Gao, et al.^52^.

#### Clinical symptom measures

The sociodemographic, clinical, and psychological characteristics of the participants were assessed using an online questionnaire through the REDCap electronic data capture software^53^. Disease duration and tender point count were evaluated by the study physician during the physical examination. Pain Duration was assessed for each individual with FMS in years.

##### Pain severity and pain interference

The severity and interference of the pain experienced were estimated using the corresponding subscales of the German version of the West Haven-Yale Multidimensional Pain Inventory (MPI-D)^54^. Both subscales are rated on a 7-point Likert scale and the scores range from 0 to 6. The final values are derived from calculating the average score. Pain severity was calculated from 3 items covering current pain, average pain over the past week, and the degree of suffering that was induced by the pain. The mean score for pain interference was calculated from 10 items investigating interference with daily life activities such as work, leisure activities or social contacts. Cronbach’s alpha was α = 0.89 for pain severity and α = 0.96 for pain interference.

##### Widespread pain index (WPI)

The extent of pain was evaluated using the Widespread Pain Index (WPI)^55^. Participants were presented with 19 potential sites on their body and asked to indicate which ones caused pain within the past 7 days. The overall score for the WPI is the total number of identified painful sites, with a range of 0 to 19. Cronbach’s alpha was α = 0.88.

##### Tender points

To assess individual myofascial tenderness, tender points were counted according to the 1990 ACR criteria^55^. Therefore, 18 possible tender points were assessed and summed up (range of 0 to 18) by a tenderness examination as part of the initial clinical assessment by the study physician.

##### Somatic symptom burden

The Somatic Symptom Burden was evaluated using the 8-item Somatic Symptom Scale (SSS-8)^56^. This scale consists of eight questions that measure the severity of pain in several regions, including abdominal pain, back pain, pain in limbs and joints, headaches, chest pain or shortness of breath, dizziness, fatigue or feelings of depleted energy, and sleep disturbances. Participants rate their symptoms on a 5-point Likert scale, ranging from “*0 - Not at all*” to “*4 - very strongly*”. Sum scores with a range of 0 to 32 were calculated for analysis, higher scores indicate greater symptom burden. Cronbach’s alpha was α = 0.84.

##### Bodily distress

To assess the degree of psychological distress caused by somatic disorders, the Somatic Stress Disorder (SSD12)^57^ questionnaire was used. With a total of 12 questions, the SSD12 encompasses psychological criteria across three subscales: affect, behavior, and cognition, with four items for each subscale. These items are rated on a 5-point Likert scale from “*0 = never*” to “*4 = very often*”. For the analysis, sum scores with a range of 0 to 48 were calculated, higher scores indicate higher distress. Cronbach’s alpha was α = 0.94.

##### Polysymptomatic distress scale

The polysymptomatic distress scale (PSD)^58^ was utilized to assess the impact of fibromyalgia, with the focus on the spectrum of multisymptomatic distress, which is also described by the term fibromyalgianess^59, 60^. This scale comprises two components: the Widespread pain index (WPI, ranging from 0 to 19) as described before and the symptom severity scale (SSS, ranging from 0 to 12). The SSS measures not only the severity of pain but also assess sleep disorders and cognitive problems. Combining these scales, a total sum score ranging from 0 to 31 is created, with higher scores indicating a more severe symptom burden^58^. Severity categories are as follows: scores between 0 and 3 indicate no severity, 4–7 indicate mild severity, 8–11 indicate moderate severity, 12–19 indicate high severity, and 20–31 indicate very severe severity. This comprehensive assessment allows healthcare professionals to evaluate and monitor the severity of fibromyalgia symptoms in patients.

### Statistical analysis

#### Data preprocessing

Study population, outcomes and main analyses were defined a priori in a statistical analysis plan (OSF.IO/G5DU7). Before the analysis, the data were screened for plausibility of the parameters, out-of-range values, and univariate outliers. This was done by calculating descriptive parameters such as mean and standard deviation, minimum and maximum values, median and the first and third quartile. A missing data analysis was performed for each variable. If missing data occurred, multiple imputation was used to handle the missings in relevant outcomes. This was the case for missing cortisol values. These were substituted by multiple imputation with *m* = 100 using predictive mean matching with the mice package^61^. All data were tested for normal distribution using Shapiro-wilk tests, skew, and kurtosis, and were visually checked for normal distribution using histograms and QQ plots. Furthermore, an outlier analysis was performed, screening for extreme outliers. Any values above or below the cutoff value of the third quartile plus/minus three interquartile distances were defined as extreme outliers and winsorized. Cortisol data were logarithmically transformed to handle extreme outliers and provide normal distribution.

#### Statistical models

For the comparison of each outcome between the two groups (individuals with FMS vs. pain-free controls), an analysis of variance (ANOVA) was performed using mi.anova() function from the *miceadds* package^62^. Additionally, a two-way analysis of covariance (ANCOVA) was conducted to control for effects of age, sex, and BMI. Further, a pearson correlation analysis was performed to investigate the associations between the outcomes as such, and the associations with clinical characteristics. These included the Widespread Pain Index (WPI), tender points, Somatic Symptom Scale (SSS-8), Somatic Stress Disorder (SSD12), Pain severity, Pain interference from MPI-D and Pain duration. Multiple comparison was handled using Bonferroni-Holm correction^63, 64^. Statistical parameters for ANOVA on imputed data were aggregated accordingly^65, 66^. Cohens *d* and partial eta were used as effect sizes, with *d* = 0.2 as small effect, *d* = 0.5 as medium effect and *d* = 0.8 as large effect, whereas the cutoff values for partial eta η_p_^2^ were η_p_^2^ = 0.01 as small effect, η_p_^2^ = 0.06 as medium effect and η_p_^2^ = 0.14 as large effect^67^. Furthermore, the correlation coefficient *r* was used as effect sizes for the correlation analysis, with cutoff values of *r* ≥ .2 as small, *r* ≥ .4 as medium and *r* ≥ .6 as large effects^67^. A p-value < 0.05 will be considered statistically significant. The statistical analysis was performed using R 4.1.2 ^68^.

## Results

### Descriptive statistics

A total of 101 individuals with FMS and 50 pain-free controls were eligible for this secondary data analysis. Two individuals with FMS were excluded due to cortisone intake, so a total of 99 individuals with FMS and 50 pain-free controls were included in the analyses. There was no significant difference in the age between the two groups (*t*(147) = 1.82, *p* = .07, *d* = 0.31). The BMI significantly differed between the groups (*t* (147) = 3.57, *p* < .001, *d* = 0.62), with higher BMI values for the FMS group (see Table 1). Both groups were characterized by a higher proportion of females. In 31.68% of the individuals with FMS pain existed for more than 20 years, in 22.7% pain existed between 10 and 20 years, in 15.84% pain was present since 5 to 10 years, 23.76% were suffering from pain between one and five years, and in 3.96% pain existed for less than one year. Group differences were found in all clinical variables such as spatial extent of pain, somatic symptom burden, bodily distress, tender points, pain severity, pain Interference, polysymptomatic distress and pain duration, as well as sick leave, with *p* < .001 and greater values for the FMS group. Detailed descriptive statistics can be found in Table 1.

**Table 1:** Descriptive statistics.

Variable	FMS (*n* = 99)	Con (*n* = 50)
Age, M (SD)	49.51 (13.08)	45.2 (14.62)
Females, % (n)	87.9% (87)	64% (32)
Sick leave in days, M (SD)	22.04 (32.21)	2.96 (5.11)
Working status, % (n)		
employed unemployed retired	77.77%	86%
	5.05%	0%
	17.17%	14%
BMI (kg/m^2^), M (SD)	27.35 (6.47)	23.82 (3.72)
Spatial extent of pain, M (SD)	11.82 (3.04)	0.64 (1.06)
Somatic symptom burden, M (SD)	16.97 (5.54)	3.08 (3.67)
Bodily Distress, M (SD)	25.76 (9.65)	3.1 (5.06)
Tender Points, M (SD)	11.61 (4.65)	0.34 (0.89)
Pain Severity, M (SD)	3.56 (0.99)	0.30 (0.61)
Pain Interference, M (SD)	3.42 (1.25)	0.30 (0.76)
Polysymptomatic Distress Scale, M (SD)	19.47 (4.07)	2.06 (2.22)
Pain Duration (years), M (SD)	16.08 (13.2)	
Perceived Stress, M (SD)	20.81 (6.65)	11.62 (6.72)
Salivary Cortisol in ng/ml	2.84 (1.69)	3.05 (1.91)
Hair Cortisol in pg/mg	8.81 (13.53)	11.55 (23.26)

*Note. M* = mean, *SD* = Standard deviation, n = Sample size, BMI = Body Mass Index, Spatial extent of pain = Widespread Pain Index, Somatic symptom burden = SSS-8, Bodily Distress = Somatic Symptom Scale (SSD-12). Salivary cortisol levels represent the daily average cortisol (DAC). Salivary and Hair cortisol levels represent the values before log-transformation.

### Group differences

**Figure 1:**
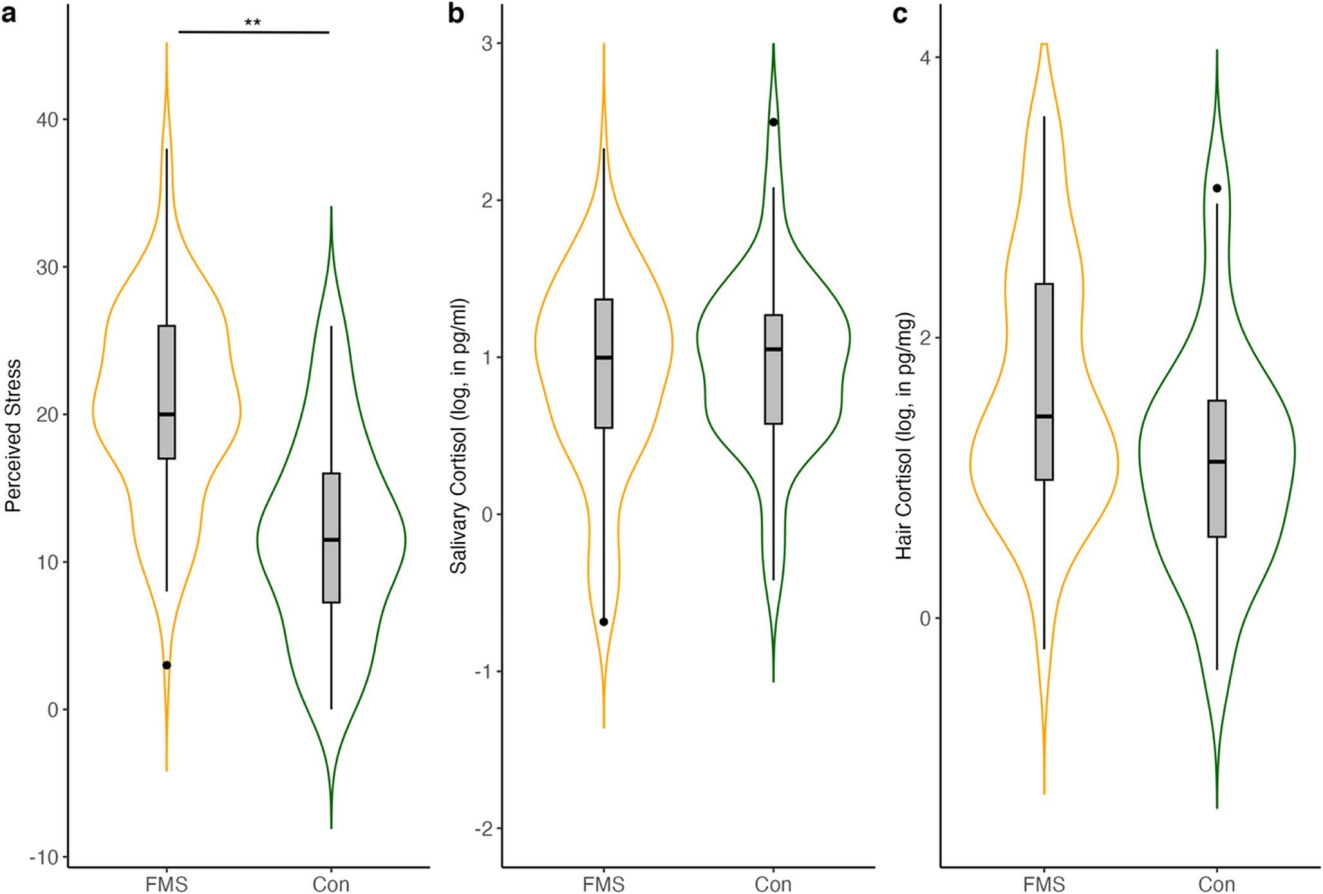
Violin Plots showing group differences of stress indicators (**a**) perceived stress , (**b**) log-transformed daily average salivary cortisol (DAC) and (c) log-transformed hair cortisol. The boxplots represent the distribution of the stress dimensions, with the mean (straight middle line) and the upper and lower interquartile ranges (1.5-fold). Points outside the boxplots represent outlier. FMS = Fibromyalgia Syndrome, Con = Controls. *** *p* < .001 .

For the analysis of group differences of the three stress dimensions PSS, salivary cortisol and hair cortisol, an analysis of variance (ANOVA) was performed in a first step. The results showed that there is a significant difference in subjectively perceived stress between individuals with FMS and pain-free controls, with *F*(1,147) = 63.03, *p* < .001, η_p_^2^ = 0.30. Individuals with FMS demonstrate higher perceived stress compared to pain-free controls. For salivary cortisol, no significant difference between the groups was found (*F*(1,63367) = 0.52, *p* = .470, η_p_^2^ = 0.004). The same applied to group differences on hair cortisol *F*(1,8427) = 0.28, *p* = .596, η_p_^2^ = 0.003. Group differences are shown in Fig. 1.

To explore whether the observed differences might be explained by covariates, a two-factor ANCOVA with the covariates sex, age, and BMI was performed. The total model for perceived stress showed significant results (*F*(1,143) = 23.34, *p* < .001, η_p_^2^ = 0.14), indicating significant group differences. The covariates sex and BMI did not have a significant influence on the model, but age demonstrated a significant influence on perceived stress (*F*(1,143) = 5.57, *p* < .05, η_p_^2^ = 0.046), with older individuals reporting lower stress levels (β = − 0.09). For salivary cortisol and hair cortisol, the covariates did not demonstrate any significant effects on the group differences.

In addition to the average cortisol parameter, we also examined the cortisol awake response (CAR) and the area under the curve (AUC) to better understand the validity of the results and the influence of different evaluation methods, as these are other common methods for the analysis of salivary cortisol^23^. None of the two parameters showed significant differences between groups, neither CAR *F*(1,139) = 1.52, *p* = .217, η_p_^2^ = 0.010, nor AUC *F*(1,129) = 0.71, *p* = .399, η_p_^2^ = 0.006.

### Associations of stress dimensions and clinical symptoms

**Figure 2:**
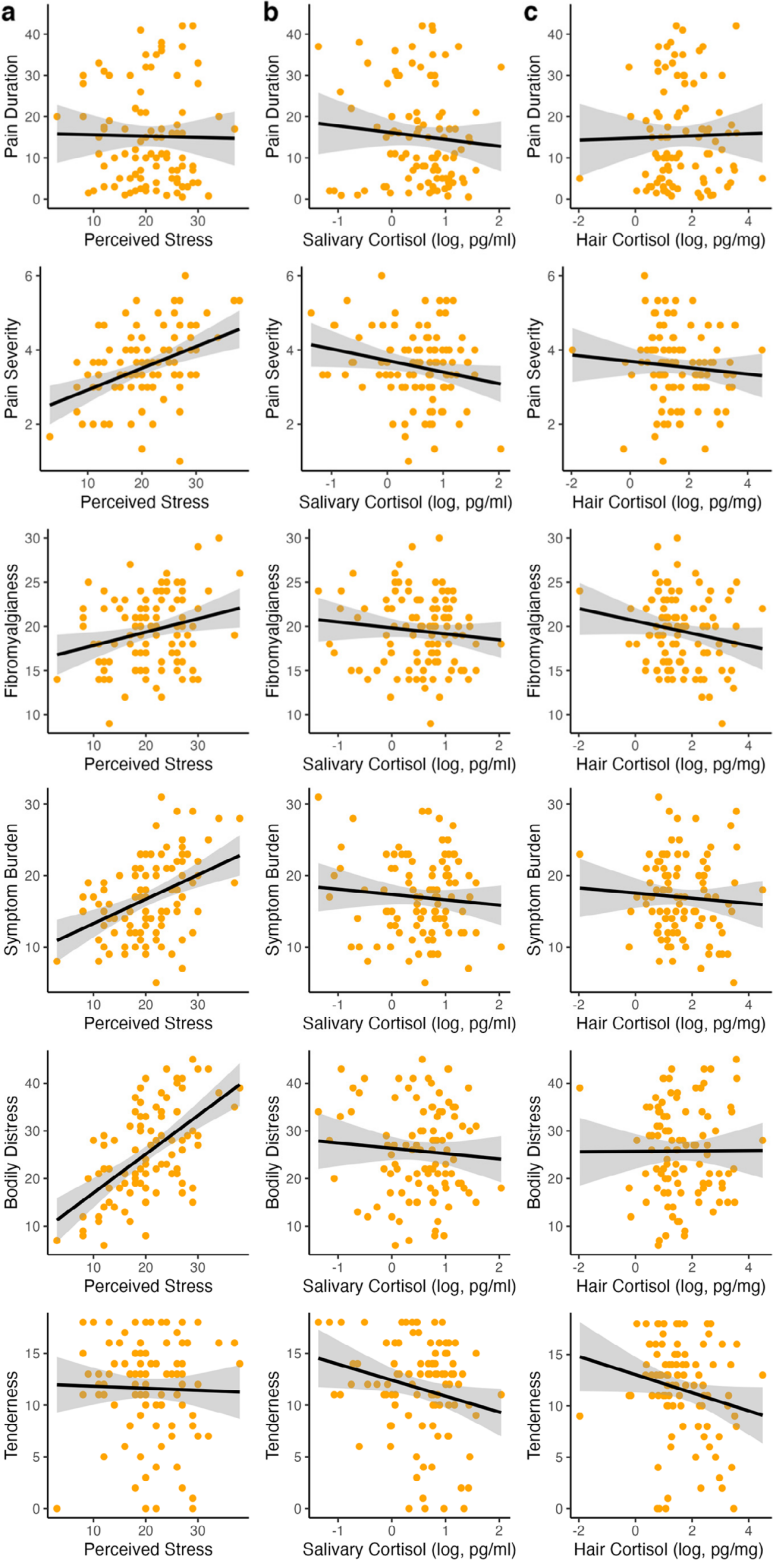
Correlations between the three stress indicators (**a**) perceived stress, (**b**) log daily average salivary cortisol (DAC) and (c) log hair cortisol with clinical outcomes for the FMS group.

For the examination of the associations among the stress indicators and clinical symptoms, a correlation analysis was performed. Significant associations were found between perceived stress and somatic symptom burden, bodily distress, pain severity, and pain interference. Overall, higher perceived stress is associated with greater symptom severity. Additionally, we found positive associations between the polysymptomatic distress scale and perceived stress. No significant association were found between salivary and hair cortisol and any of the clinical outcomes. A negative significant correlation was found between age and salivary cortisol, indicating, that the older the sample, the lower the salivary cortisol levels. Furthermore, no significant relationship between the stress dimensions themselves were found. Detailed results for the correlation analysis are available in Table 2; Fig. 2.

**Table 2:** Correlation coefficients of stress indicators for the FMS sample *N* = 99.

Variable	Perceived Stress	Salivary Cortisol	Hair Cortisol	Age	BMI	Symptom Burden	Bodily Distress	Spatial Extent of Pain	Tender Points	Pain Severity	Pain Inter-ference	Pain Duration
Perceived Stress												
Salivary Cortisol	0.13											
Hair Cortisol	0.17	0.06										
Age	− 0.06	− 0.25	0.01									
BMI	− 0.03	− 0.14	0.07	0.10								
Symptom Burden	0.41**	− 0.08	0.03	0.03	0.14							
Bodily Distress	0.56**	− 0.06	0.03	− 0.04	0.02	0.46**						
Spatial Extent of Pain	0.03	− 0.13	− 0.11	0.17	0.13	0.29	0.10					
Tender Points	− 0.02	− 0.18	− 0.09	0.32	0.29	0.44**	0.13	0.43**				
Pain Severity	0.39**	− 0.19	− 0.06	0.13	0.19	0.48**	0.41**	0.06	0.28			
Pain Interference	0.55**	− 0.10	0.01	0.00	0.17	0.48**	0.61**	0.11	0.17	0.72**		
Pain Duration	0.05	− 0.13	0.04	0.39**	0.09	− 0.03	− 0.07	0.07	0.11	− 0.05	0.00	
Fibromyalgianess	0.25	− 0.12	− 0.08	0.10	0.02	0.50**	0.18	0.82**	0.44**	0.22	0.23	0.09

Note. *p < .05, ** *p* < .001. Perceived Stress = Perceived Stress Scale (PSS), BMI = Body Mass Index, Symptom Burden = Somatic Symptom Scale (SSS-8), Bodily Distress = Somatic symptom Disorder (SSD12), Spatial Extent of Pain = Widespread Pain Index (WPI), Fibromyalgianess = Polysymptomatic Distress Scale (PSD).

### Exploratory data analyses

Additional exploratory analyses were performed to investigate subgroups of individuals with FMS regarding the stress indicators and whether there are subgroups of high or low cortisol profiles in individuals suffering from FMS. The FMS group was therefore divided into six subgroups with (1) high or (2) low salivary cortisol levels, and (3) high or (4) low hair cortisol levels and another two groups, one characterized by (5) high salivary and high hair cortisol and the other one characterized by (6) low salivary and low hair cortisol, using median split. These analyses revealed negative associations of the low-salivary cortisol group (*n* = 31) between salivary cortisol and bodily distress (*r* = − .355, CI_95_ [-0.63, -0.00]) and pain severity (*r* = − .42, CI_95_ [-0.67, -0.08]), and negative associations of the high-hair cortisol group (*n* = 42) between hair cortisol and pain duration (*r* = − .323, CI_95_ [-0.57, -0.02]). In the group with low salivary and low hair cortisol (*n* = 16), a negative association was found between pain severity and salivary cortisol (*r* = − .52, CI_95_ [-0.81, -0.04]).

We further calculated exploratorily the increase in cortisol awakening response (CARi), which revealed CARi_1_ = 1.11 and CARi_2_ = 0.91 on day 1 and day 2, respectively. CARi_1_ for the control group was 0.97 and CARi_2_ = 0.90, respectively. Salivary cortisol group profiles including CARi are represented in Appendix D in the supplementary material.

## Discussion

The primary aim of this study was to explore potential differences in stress levels between individuals with FMS and pain-free controls across several stress dimensions, including both perceived stress and endocrine indicators of stress. Stress assessment included three different dimensions: perceived stress levels, and daily average salivary cortisol and hair cortisol concentrations as indicators of acute and chronic stress levels via the HPA-axis. This comprehensive approach allowed us to identify and compare stress indicators across a broader spectrum. The main findings of this study were that (1) individuals with FMS reported significantly higher levels of perceived stress compared to pain-free controls, while (2) the cortisol data did not show significant group differences. Furthermore, correlation analysis showed that (3) clinical symptoms were correlated closely with perceived stress but not with cortisol indicators. No significant associations were observed between the stress dimensions themselves.

The finding that perceived stress in the last month was higher in individuals with FMS compared to controls, while there is no evidence on differences on cortisol markers of stress, is partly in line with the literature. As several meta-analyses have shown in recent years the evidence on cortisol levels in FMS is still very inconsistent and controversial^21, 32, 33^. For example, several studies found no significant differences in hair cortisol concentrations between individuals with somatic functional disorders, including FMS, and healthy controls^36–38^. Further, similar to our data, Fischer, et al.^36^ also found no association between self-reported stress and hair cortisol. In addition, Coppens, et al.^31^ found no significant differences in baseline salivary cortisol, but significant differences in perceived stress between individuals with FMS and healthy controls. However, these results are in contrast to other studies that reported positive associations between hair and salivary cortisol^69^ or hair cortisol and perceived stress^37, 70^, although effects were weak. Despite several studies in the literature have already reported inconsistent findings regarding deviations in HPA-axis activity in FMS, recently the connection between cortisol levels and symptom severity was highlighted with introducing a model that links hair cortisol concentrations with both disease duration and pain intensity^37, 38^. In this context, the absence of significant differences in cortisol levels between FMS patients and controls in our study might be partially explained by the more homogeneous nature of our sample regarding disease duration. In the two-component model by Reyes del Paso, et al.^37^, which included a smaller sample than in our study, significantly higher overall perceived stress levels were reported for both FMS and healthy controls compared to our population. While effects can vary with different sample sizes, and both studies maintain high qualitative standards, the variability in findings emphasizes the need for future research with larger samples to clarify these inconsistencies. The proposed model by Reyes del Paso, et al.^37^ suggests that cortisol levels may vary considerably depending on the stage of the disease and symptom severity, which our study did not explicitly find, however, is suggested by exploratory subgroup analyses. Future research should incorporate such models to better capture the dynamic nature of the HPA-axis in FMS, potentially revealing subtler physiological changes associated with chronic stress in this population.

Research in this area presents challenges that can significantly impact study results. Studies on salivary cortisol use different designs, sample numbers, and assay methods, leading to heterogenous outcomes^22, 23^. Hair cortisol is seen as a more stable long-term marker for chronic stress^19^ due to some advantages over salivary cortisol, but factors like hair washing frequency or physical activity can affect the results^25, 43, 71, 72^. It is unlikely that a single stress measure can fully capture the activity of the body’s stress response system, as there is a complex interplay between multiple biological systems.

Our results show no significant associations between cortisol indictors of acute and chronic stress levels and clinical symptoms. However, when interpreting these data, it should be borne in mind that our cortisol measures only represent the systemic cortisol response, particularly that of the HPA-axis. We did not examine factors such as the sympathoadreno-medullary (SAM) axis or the neuroimmunological stress response. Therefore, we cannot extend our results to indicators of stress system activity beyond HPA activity. However, the SAM-axis and the body’s inflammatory system and their interaction with the brain’s neuronal networks play an important role in coping with stress^73^.

In this regard, the imbalance of threat and soothing systems theory of stress by Pinto, et al.^3^, as well as the generalized unsafety theory^13^ offer interesting concepts. Here the interaction of bodily cues, impaired interoception, challenging social contexts and the potential amplification of these factors by acute and chronic stress are emphasized. This multifaceted perspective underscores the critical importance of addressing stress, both acute and chronic, in the assessment and treatment of FMS. Psychological factors consequently play a crucial role in the perception and handling of pain, and stress in turn is associated with such psychological features. Internal and external control, for example, have relevant influences on how patients may cope and handle their pain in everyday life and how they may respond to treatments^28, 74–76^. It is important to recognize that the subjective experience of stress results from the interconnection of all these components. In parallel, clinical symptom burden is strongly influenced by daily experiences that are embedded in a neural network of the brain that includes emotional and evaluative aspects that may lead to a more sensitive response to stress and pain^3^. Thus, consideration of individual markers of HPA activity alone is not sufficient to describe the stress response in individuals with FMS.

Even though no significant differences were found at the overall group level, this does not mean that the HPA-axis is completely uninvolved in the complex interplay of FMS pathophysiology. While the main results of our study suggest a dissociation of the examined indicators, clinical correlations for cortisol were found in exploratory subgroup analyses. Subgroups of individuals with FMS were identified based on their cortisol profiles, including high and low salivary or hair cortisol groups, and combinations of high and low cortisol on both measures. These subgroups showed different associations with clinical outcomes such as physical distress, pain severity, pain duration and pain interference. While the exploratory nature of the findings limits interpretation, it could suggest that there may be an association with clinical symptoms, particularly in certain subgroups and in cases of extreme HPA-axis dysregulation. Interestingly, our findings on group differences in perceived stress revealed not only that the FMS group showed greater perceived stress, but also that the covariate age had a significant influence, with older individuals showing less perceived stress. This effect may be because individuals suffering from pain learn different coping strategies over time and gain more experience in handling pain and stress in everyday life. Further, with increasing age, patients have experienced more different psychotherapeutic approaches and learn to accept their pain, even though therapies are often not sufficient in treating chronic pain^77, 78^. Our findings align with those of Wettstein, et al.^79^, who found a “paradoxical” model of age-related effects in chronic low back pain patients. Similar to our findings, they observed that the quality of life remained the same, or improved with age, although disability worsens with older age. This suggests that older individuals may develop better coping mechanisms and resilience over time, which could explain the reduced perceived stress observed in our study. Another explanation may be that the biological stress system adapts in the long-term^38, 41, 80^. In our correlation analysis we found a negative association between age and salivary cortisol. Further, the findings of our exploratory subgroup analyses showed inverted relationships between salivary cortisol and pain severity, as well as between hair cortisol and pain duration. These results are in line with the results of Reyes del Paso, et al.^37^, who reported higher hair cortisol concentrations in patients that reported a shorter duration of FMS. Hence, these results may point to the adaption of the body’s stress system over time, indicating a less intense reaction of the stress system by increasing chronicity. The duration of pain is a crucial aspect in the development of stress reactions. It is assumed that there are different subgroups in chronic pain patients, characterized by different factors. For example longer pain and enhanced stress chronicity is assumed to lead to hypocortisolism^37, 41, 81, 82^, whereas in contrast hypercortisolism rather occurs in individuals that have been newly affected by FMS and suffer from more acute stress^32, 42, 80, 83^. Consequently, the duration of the disease and the symptom severity are crucial factors as subgroup analyses indicate, but also the exposure to adverse events leading to chronic stress need to be considered. Therefore, it is important to assess these factors more precisely to investigate biological adaptions in terms of chronic stress and chronic pain accordingly. However, it is important to emphasize that our results of the exploratory data analyses with small sample sizes should be interpreted with caution and further studies are needed to support those findings, before jumping to conclusions. Next to this point, further research should also focus on the development of clinical approaches targeting subjectively perceived stressors, together with a broad investigation of a wide range of biomarkers using of multi-omics approaches to determine phenotypes of the HPA-axis to investigate the biological network underlying FMS, to clarify and better understand the pathophysiology.

## Limitations

Some limitations need to be mentioned. Next to unequal samples sizes of our compared groups, sex was not equally distributed. However, the adjustment for sex did not reveal significant influences on the results. Further, hair cortisol was not controlled for influencing factors such as hair washing frequency, shampooing or hair coloring. This could potentially lead to subtle differences being overlooked that were not detectable within the scope of our analysis. Neither did we control for physical activity, nor for the menstrual cycle of the participants. The data were collected during the COVID-19 pandemic, which was a stressor that had effects on physical and mental health and human behavior. This may have weakened the contrast between FMS and controls, thus weakening the power of our analyses. In addition, the time periods that the three stress indicators aim to cover only partially over-lapped, making it possible that stress exposures could fall within the time frame of one indicator but outside the recording period the others. This limits the interpretation of the results and underlines the question whether the chosen indicators were sufficient to represent acute and chronic stress. In such cases, experience sampling methods may provide better approaches to collect data on perceived stress while collecting biological stress measures. These limitations may restrict the interpretation of findings, however, prescribed standards for the analysis and interpretations were applied.

## Conclusion

In our study of FMS individuals and pain-free controls, individuals with FMS reported significantly higher subjective stress levels, closely related to symptom severity. Importantly, a dissociation between perceived stress and cortisol indicators of stress was observed. We found no evidence linking FMS to HPA axis-related markers of acute and chronic stress levels like cortisol concentrations in saliva or hair. This underscores the need for nuanced clinical approaches that target perceived stress in individuals with FMS to improve symptom management and to reveal the complex relationship between stress perception and physiological stress responses.

## Electronic supplementary material

Below is the link to the electronic supplementary material.

Supplementary Material 1

## Data Availability

Due to local data protection regulations, direct release of the data in the sense of accessibility is not possible. The possibilities for data availability must be clarified with the corresponding author on request in individual cases.
