# Supplementary material for: Perceived and endocrine acute and chronic stress indicators in fibromyalgia syndrome
Source: Sci Rep. 2024 Dec 16;14:30471. doi: 10.1038/s41598-024-76635-z (PMC11649902; doi:10.1038/s41598-024-76635-z)
Supplement: Supplementary file 1 — Supplementary Material 1 [file 41598_2024_76635_MOESM1_ESM.docx]

**Supplemental Material**

Perceived and endocrine acute and chronic stress indicators in fibromyalgia syndrome

Eva Beiner^a^, Michelle Hermes^a^, Julian Reichert^a^, Kristian Kleinke^b^, Stephanie Vock^a^, Annette Löffler^c,d,e,q^, Leonie Ader^f^, Andrei Sirazitdinov^g,h,i,j^, Sebastian Keil^c^, Tim Schmidt^f^, Anita Schick^f^, Martin Löffler^c,k^, Michael Hopp^l^, Christian Ruckes^l^, Jürgen Hesser^g,h,i,j^, Ulrich Reininghaus^f,o,p,m^, Herta Flor^c^, Wolfgang Eich^a^, Hans-Christoph Friederich^a,m^, *Jonas Tesarz^a,m^

**Content**

**Appendix A. Flow Chart**

**Appendix B. PerPAIN procedure outline**

**Appendix C. Correlation analyses for the total sample (N = 149)**

**Appendix D. Salivary daily cortisol profiles**

**Appendix A. Flow Chart**

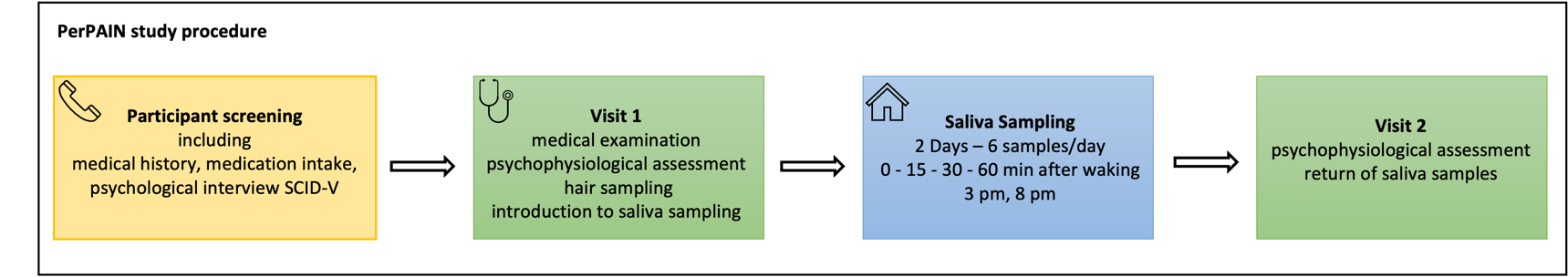
**Appendix B. PerPAIN procedure outline**

*Note. PerPAIN study procedure*

**Appendix C. Correlation for the total sample N = 149**

Note. Correlations between the three stress indicators (a) perceived stress, (b) log salivary cortisol and (c) log hair cortisol with clinical outcomes.

Green = pain-free controls, yellow = FMS


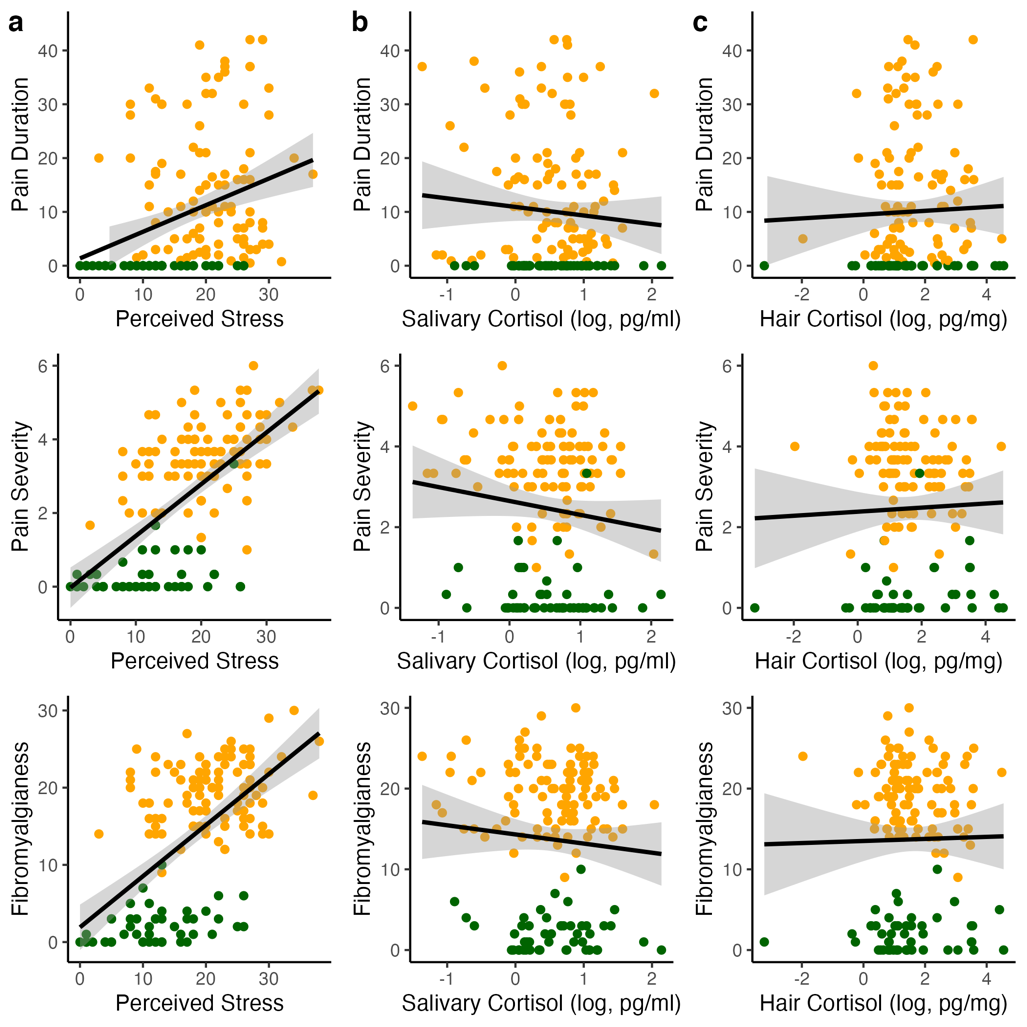

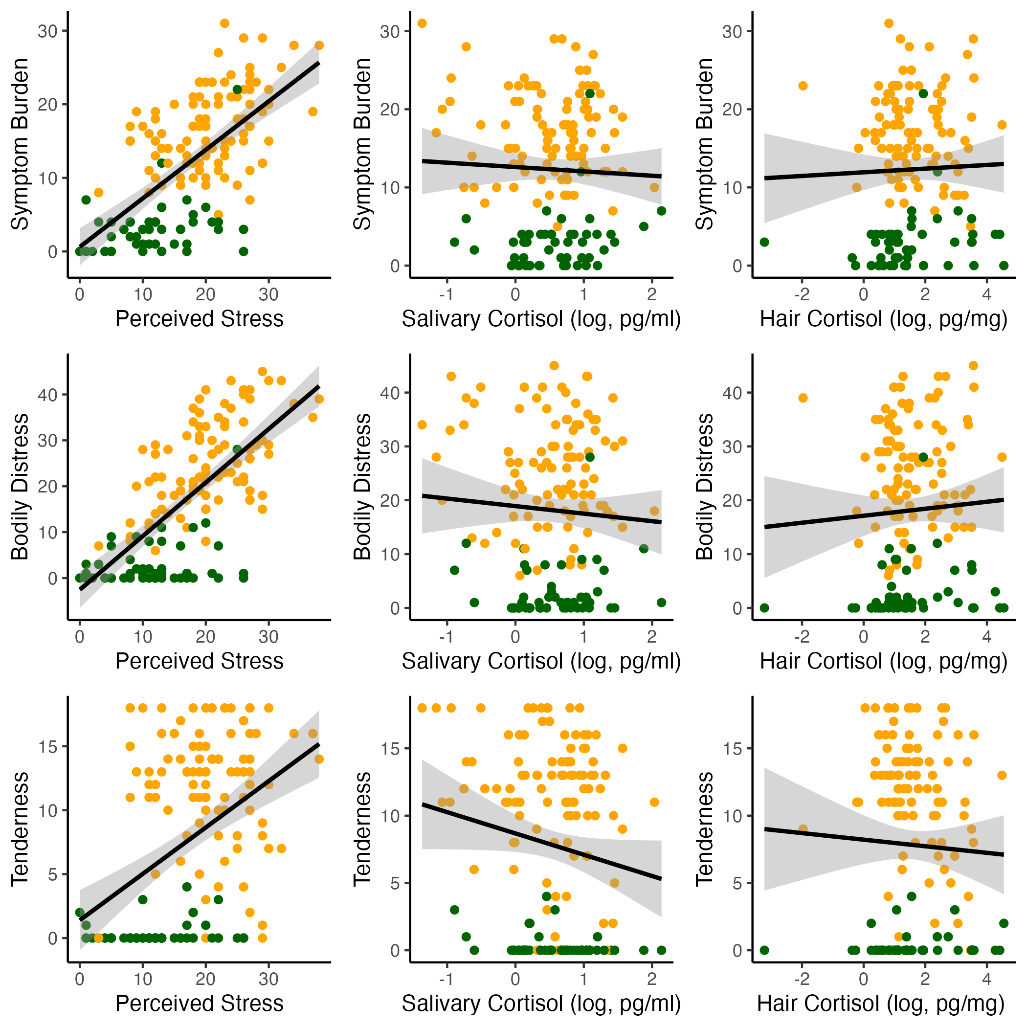


**Appendix D. Salivary daily cortisol profiles**


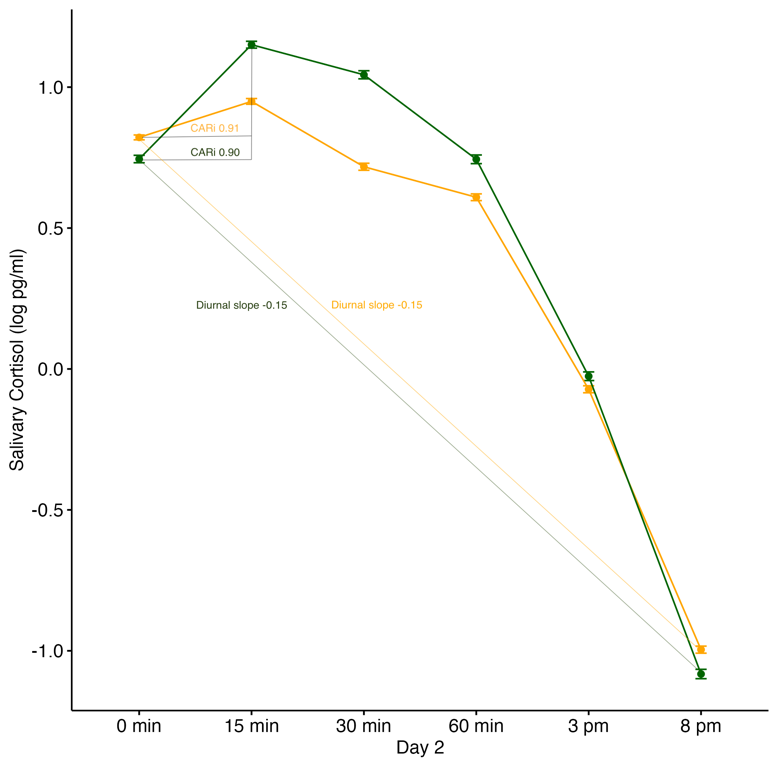

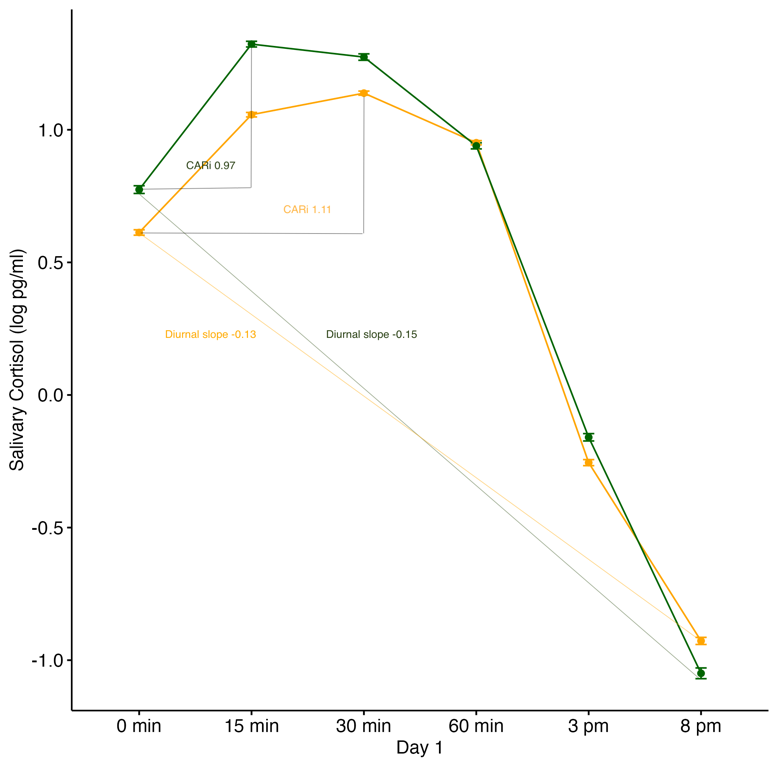


*Note. Salivary cortisol profiles for day 1 (left) and day 2 (right), with increase in cortisol awakening response (CARi) and diurnal slopes. Yellow = FMS, Green = Controls.*
